# Clinical Studies with Non-Nauseating Doses of Ipecacuanha, Chiefly in Intermittents

**Published:** 1875-06

**Authors:** Alfred A. Woodhull

**Affiliations:** Assistant Surgeon U. S. Army


### CLINICAL STUDIES WITH NON-NAUSEATING DOSES OF IPECACUANHA, CHIEFLY IN INTERMITTENTS.

By ALFRED A. WOODHULL, M.D., Assistant Surgeon U. S. Army.

*An Abstract from a Special Report to the Surgeon General U. S. Army, read before the Atlanta Academy of Medicine.*

In a recently published paper (Atlanta Medical and Surgical Journal, February–May, 1875,) upon the use of large non-emetic doses of ipecacuanha in certain intestinal affections, I ventured two or three hypotheses. The most important of these is that that drug is a nervous stimulant, especially as to the ganglionic system, and is therefore indicated non-emetically in the correction of various trains of symptoms that may follow a depressed condition of the sympathetic. It was also suggested that, as that system may be intimately involved in the paroxysmal fevers, ipecacuanha may prove useful in them; and, while certain well-known practice in that direction was referred to, further observations were called for. As a contribution to the subject, the following is offered as so much clinical material. To restrain this paper within reasonable limits I shall not be able to detail all my experience, but I invite attention to the particulars as far as given, and to the summary. The cases were treated experimentally.

The object steadily kept in view was an examination of the influence of ipecacuanha over malarial poisoning, and therefore neither adjuvants nor substitutes were used, nor was uniformity in the doses preserved.

Aware of the tendency among soldiers to avoid duty by feigning the lighter forms of sickness, especially chills, only those cases have been taken account of that I have personally watched or which were observed by reliable third parties, and whatever rests on the statement of the men themselves is so noted. Most of the earlier cases were drawn from a detachment that was stationed in a highly malarious region in Alabama from the middle of September until the latter part of November, 1874, where nearly all of the command suffered severely with that poison. Atlanta, whither they returned late in November, is high and breezy in location, with granitic subsoil, where the paroxysmal fevers rarely originate. I am assured, however, by prominent resident physicians that imported cases are exceedingly obstinate and show little or no tendency to spontaneous cure. My own experience agrees with this. Very few of the cases were continued ague that might be supposed to have yielded to climatic influences, but the most, and especially the severer ones, were attacks that recurred after temporary suspension by quinine.

Case I.—J. S. rejoined this post from Alabama 25th November, 1874. He was seriously sick there for nearly two months with a fever that finally took the form of tertian intermittent, which lasted three weeks. One of these chills occurred on 27th, and he was taken in hospital with the view of trying ipecacuanha in this affection.

28th.—℞. Pulv. ipecac., gr. j, *ter die.* Temperature: 10 A.M. 101; 3 P.M. 100 2-5; 6 P.M. 100 4-5. Notwithstanding this excess he did not recognize it by his sensations.

29th.—This was the day for the chill, and about the hour it was due there were some indications of it, but there was no rigor and no fever. Temp. A.M., 99 2-5; P.M., 100 2-5.

The same treatment, namely, one grain of ipecacuanha three times a day, was continued while he remained on sick report, and the following are the thermometrical notes: 30th, A.M., 98 4-5; P.M., 98 3-5: 1st Dec., A.M., 97 4-5; P.M., 98 3-5: 2d, A.M., 98 4-5; P.M., 98 1-5: 3d, A.M., 98. Duty.

The first dose on the first day caused merely a little nausea, which did not happen again. No appearance of chill occurred after 29th. The medicine was persevered with a week after his return to duty. On the fifth or sixth day he had a slight headache, but no chill, and the ipecacuanha was continued another week. The very prompt cessation of the ague following the change of locality would throw doubt upon the influence of the medicine if this case stood alone.

CASE II.—C. S. E. returned from Alabama on the night of 30th November, 1874. The attending medical officer gave this history of him. Seven or eight weeks ago he was attacked with well-marked tertian intermittent, for which he took sixteen grains of quinine. This stopped the chills, and for a week he took pills each containing two grains of quinine and sub-carbonate of iron and one-thirtieth of a grain of arsenic. He ceased taking this, contrary to orders, and the ague returned in a few days. Sixteen grains of quinine again suppressed it, and he continued taking the pill above described until 25th November, when he had a well-marked chill. On 26th he took twenty-four grains of quinine and, the company marching, received no more medicine.

5th December.—Reported at sick call, asserting that he had had a severe chill, lasting two hours, at three o'clock the previous afternoon, followed by a fever for several hours. Admitted to hospital at once, and given one grain of ipecacuanha every every six hours. Temp., 7:30 A.M., 97 3-5; 2:30 P.M., 97 4-5; 4 102 1-5; 8:30 101 4-5. This was not the chill day, but he had a light chill from 4 to 4:30 P.M., not recognizing any fever.

6th.—To take one grain every four hours to-day. Temp., 7 A.M., 98; 1 P.M., 98; felt well till 4 P.M., when a light chill, lasting between thirty and forty minutes, occurred. Temperature during the chill 98 2-5 (this was carefully observed); at the beginning of the fever 103 2-5; 6 P.M., 104 1-5; 8 P.M., 103 1-5.

7th.—Temp., 7 A.M., 97 2-5; 3 P.M., 98; 6 P.M., 98 1-5. One grain every four hours was continued without nausea.

8th.—Temp., 7 A.M., 97 1-5; 4:30 P.M., 99 1-5; 6 P.M., 98 4-5. Although this was the regular day for the chill he had no abnormal sensation, the thermometer alone indicating a derangement. He took two grains every three hours this day.

9th.—Treatment continued. Temp., A.M., 98 2-5; P.M., 99 2-5.

10th.—Treatment continued. A careful thermometrical watch was kept for the chill, but there was no indication of it, and he said that he felt better than he had for a long time. Temp., 7 A.M., 97; 9 A.M., 98; 1 P.M., 98 2-5; 4 P.M., 98 2-5; 7 P.M., 99.

11th.—Returned to duty, and directed to take one grain of ipecacuanha every six hours for three weeks, and to report any indication of ague.

11th January, 1875.—E. reports that he has taken no medicine for a fortnight, that on 9th he had a chill, and that he also had one to-day. For some time he has been daily exposed to cold and wet as a quartermaster's laborer. Given one-grain pills with directions to take two every two hours and to report promptly if not better. He also was relieved from extra-duty as a laborer and was returned to ordinary military duty.

There was no further trouble with this case, which was regarded by the officer first treating it as peculiarly obstinate and as well suited to test the antiperiodic powers of the drug.

Case III.—Lieutenant W. This officer has recently spent two months at Butler, Ala., where he found it necessary to take prophylactic doses of quinine daily. After his return here he had irregular symptoms of ague, culminating on 8th December, 1874, in a mild chill, which recurred, with some febrile reaction, on the ninth. Chills in his case are never well marked by rigors, but are always of the so-called "dumb" variety. For several years past, following severe malarial intoxication in Texas, he has been liable to such disturbances after comparatively slight provocation. His bowels are sluggish. Temp. 4 P.M. 99. ℞. Pil. cath. c., no. iij, at once. Ipecac., gr. j, every three hours.

10th.—Bowels moved freely but not excessively. The ipecacuanha induced disagreeable nausea but no vomiting; the skin became moist and he felt better, although there was some chilliness and febrile disturbance at the usual time, a little after noon. Temp. 4 P.M., 99. Continue the ipecacuanha.

11th.—Less nausea; skin moist; not chilly but is disinclined to move much in the open air; bowels loose but not purging; temperature not noted. To take after to-day one grain every six hours.

12th.—Not so well as yesterday; more chilly but not regularly aguish; better in the afternoon; temp. 4 P.M., 100. To take one grain every three hours and two grains at 12 M. to-morrow.

13th.—Much better; no chilliness.

This treatment was kept up for several weeks, with gradually increasing intervals between the doses. He continued to steadily improve, so that, although he had at no time been off duty from sickness, his better health was plainly observed and commented upon by himself and others.

The following is the only case in my experience, to the present time, where the disease did not promptly yield to ipecacuanha. It is not presented as a model for imitation, but to illustrate the less favorable phases of the treatment, and because it may be useful as a collection of clinical notes. In this was the first departure from the use of small doses in intermittents.

CASE IV.—J. S. In August, 1874, hypodermic injections of quinine were used with this man on account of intermittent fever. (See *New York Medical Journal*, xx., 5, p. 498.) Early in October, in Alabama, chills came on every two or three days, he says. These were checked by large doses of quinine, which he took to cinchonism. In the intervals he took some sort of bitters, but the least fatigue or exposure reinduced the disease. He took quinine on the march to Atlanta. Had a chill here 29th November, and again took quinine that he had in his possession. Was free from chills for two weeks, but he had much lassitude and weariness, and the disease reappeared. Says he had a severe chill 13th December, and that he took two three-grain quinine pills at noon and also at night.

15th December.—I saw him in the reaction of a second severe chill. He is sallow and cachectic, with the appearance of being saturated with malaria.

16th.—Admitted hospital this morning, having taken no medicine meanwhile. Temp. A.M., 98 1-5; P.M., 99 4-5. Took ipecac. gr. j every three hours from 7 A.M., and gr. ij at 8:30 P.M.

17th.—Took two grains at 1 and 6:30 A.M., when he became nauseated but did not vomit. Afterward continued gr. j every three hours. Temp. A.M., 98; P.M., 98 2-5. This was the regular day for the chill.

18th.—Temp. 7 A.M., 97 1-5; 10:30 A.M., 98 3-5; 1 P.M., 99 1-5, (evening notes lost.) Felt cool this morning. ℞. Ipecac. gr. ij every three hours and ipecac. gr. v., opii gr. ¼ at 9 P.M.

19th.—Some pain in the legs; no nausea. Temp. A.M., 98 2-5; pulse 104; 1 P.M., 98 3-5; 5 P.M., 99 2-5; p. 104. ℞. Ipecac. gr. v. opii gr. ¼ at 10 A.M. and 8:30 P.M.

20th.—Temp. 7 A.M., 98 2-5; 9:30 A.M., 99; 1 P.M., 99; 6 P.M., 99 2-5. Took ipecac. gr. v., opii gr. ¼ at 10 A.M., 2 and 8 P.M.

21st.—Temp. 9 A.M., 98 1-5; 1 P.M., 99; 6 P.M., 99 2-5. ℞. Ipecac. gr. j every two hours from 9 A.M.

22d.—Temp. A.M., 99; P.M., 99. Ipecac. gr. ij every two hours.

23d.—Temp. A.M., 98 3-5; P.M., 99 2-5. Treatment continued.

24th.—Temp. A.M., 98 3-5; P.M., 99 3-5. ℞. Ipecac. gr. v., opii gr. ¼, *ter die.* Notwithstanding these thermometrical fluctuations he insists that he feels well, excepting a loss of appetite. The cook being ill, he was put at work in the hospital kitchen. Up to this point it will be observed that he had no chill since beginning the treatment.

25th.—Temp. A.M. 99 3-5; 1 P.M., 101; P.M., 101 3-5. Treatment continued.

26th.—Temp. A.M., 98; 1 P.M., 99 4-5; 6 P.M., 100 1-5. In bed during the day with a chill. ℞. Ipecac. gr. ij every two hours.

27th.—Temp. A.M., 99 3-5; 1 P.M., 98 4-5; P.M., 100 4-5. A chill to-day. Two grains throughout the day and five at night.

28th.—Temp. A.M., 98 2-5; P.M., 99—Chill. Two grains throughout the day.

29th.—Temp. A.M., 102 1-5; P.M., 99 4-5—Chill. A profuse sweat early in the morning.

30th.—Temp. A.M., 97 3-5; P.M., 100 2-5—Chill. Treatment continued.

31st.—Temp. A.M. 97 3-5; P.M., 98 4-5—Chill. Treatment continued. It is almost certain that other, or at least additional, measures would have benefited this man during the past week, but I was anxious not to complicate the treatment. His saffron hue, and other indications of functional hepatic derangement and general depression, strongly called for supplemental remedies which, however, were not used. The chills increased in apparent severity to this date, after which they ceased except on one day.

1st January, 1875.—Temp. A.M., 97 3-5; P.M., 99 2-5. ℞. Ipecac. gr. ij every two hours. No chill.

2d.—Temp. A.M., 99 1-5; P.M., 99 3-5. ℞. Ipecac. gr. ij every two hours, and ten grains with one-half grain opium at night.

3d.—Temp. A.M., 98 4-5; P.M., 98 4-5. Treatment continued. No chill, but a cold sweat this night.

4th.—Temp., A.M., 98; P.M., 98 3-5. Treatment and condition as yesterday.

5th.—Temp., A.M., 98; P.M., 98 4 5. Treatment and condition the same.

6th.—Temp., A.M., 98; P.M., 98 4-5. Two grains every two hours. Seen this evening by Asst. Surg. Maus who, for the cold perspiration, gave acid. sulph. arom., *m.* xv, at bedtime.

7th.—Had less perspiration last night. Took two grains every two hours during the day. Temp., A.M., 97 1-5; P.M., 98 4-5.

Took, without water, ipecac. gr. xx., opii gr. j, in pil. iij, at 7:30 P.M., going to bed then. Felt sick at 10 P.M., and at 11 P.M. vomited, at two efforts, five minutes apart, in all a half-pint of bitter fluid. Had no nausea after the second vomiting, and fifteen minutes later took ipecac. gr. x., opii gr. ss., in pil. ij. Slept well the remainder of the night. Between 7:30 and 8:30 P.M. he had warm but no cold perspiration.

8th.—Temp. A.M., 98 3-5; P.M., 98 3-5. No ipecacuanha during the day, but took twenty grains at 8.30 P.M., and ten grains in the night. Was somewhat nauseated, but neither vomited nor perspired.

9th.—Temp. A.M. 98 3-5; P.M., 99 1-5. Took thirty grains at tattoo; neither vomited nor perspired.

10th.—Temp. A.M., 98 2-5; P.M., 99 2-5. Nocturnal seminal emissions annoying him, he was given tr. fer. chl., *m* xv., every six hours, and the ipecacuanha was omitted. No perspiration.

11th.—Temp. A.M., 99; P.M., 99 1-5. Yesterday's treatment continued. Had a marked warm perspiration this night.

12th.—Temp. A.M., 99; P.M., 99 2-5. Treatment continued. A slight perspiration to-night and no emissions for two nights.

13th.—Temp. A.M., 98 2-5; P.M., 99 2-5. Feels very well; appetite good; no chilly sensations for some days, but has a little cardiac palpitation.

14th.—Temp. A.M., 98; 1 P.M., 100 2-5; P.M., 99 4-5. Had a light chill about noon. Continued the iron, and gave ipecac. gr. x., opii gr. ss., 8:30 P.M.

15th.—Temp. A.M., 97 2-5. Felt well all this day. Continued iron, but omitted ipecac. Returned to quarters.

16th.—Temp. A.M., 99; 1 P.M., 99 4-5; P.M., 98 2-5. ℞. Ipecac. gr. ij every three hours.

17th.—Temp. A.M., 97 2-5; P.M., 99 1-5. Treatment continued.

18th.—Temp. A.M., 98; P.M., 99. Treatment continued. Warm perspiration for an hour and a half this night.

19th.—Temp. A.M., 97 4-5; P.M., 99 4-5. Counter-irritation ordered for a little, evidently neuralgic, costal pain. ℞. Ipecac. gr. iij, three or four times a day.

20th.—Temp. A.M., 97 3-5; P.M., 99 1-5. Treatment continued. Vomited a little at 3:30 P.M.

21st.—Temp. A.M., 97; P.M., 99 2-5. Treatment continued. Dry cups to the side.

22d.—Temp. A.M., 98 4-5; P.M., 99 2-5. Treatment continued. Chest Faradized.

23d.—Temp. A.M., 96 2-5; P.M., 100 1-5: 24th, 98 3-5; P.M., 99 3-5: 25th, A.M., 98 2-5; P.M., 99 3-5: 26th, A.M., 98; P.M., 99 4-5: 27th, A.M., 98; P.M., 100 3-5.

28th.—Up to this date the treatment described was persevered in, with gradual general improvement. To-day he passed under the charge of Asst. Surg. Maus, who prescribed four three-grain quinine pills, one to be taken every three hours, and returned him to duty. There was no relapse, and he speedily became strong and vigorous.

We find in this case four stages: (1.) An ordinary but severe intermittent, which promptly responded to the medicine in small doses. (2.) An obstinate recurrence, with increasing severity for six days, when the paroxysms suddenly ceased. (3.) A stage of cold sweating, with seminal emissions, for which iron was used. (4.) A stage of slow convalesence, complicated with neuralgic pains.

CASE V.—Attention is invited to the earlier part of this case, as illustrating the association between intermittents and acute dysentery, and the influence of ipecacuanha on both diseases.

A. S. This man gives the following history of himself, which is corroborated by his officers. Being on duty in Alabama, for the first ten days he was well. Then being on the road for three days and nights continuously, on duty as a teamster, was immediately thereafter seized with "swamp fever," with which he was ill several weeks. He then did duty for a week, when tertian intermittent set in, lasting two weeks. He then did duty for a fortnight, when the chills recurred, during which attack his company returned to this, their permanent station. He had one chill on the road but none after reaching the post, 28th November, 1874, until the attack here noted.

On 12th and 13th December he felt weak and unwell. On 14th had a light chill coming on about 2 P.M., the fever lasting until nine o'clock. On 15th had a more severe chill, which, with the fever, lasted from ten to three o'clock. 16th, no chill.

17th December.—Up to this date he discharged his duty (as teamster) and did not apply for medical assistance. Felt badly early in the morning, and about nine o'clock a painless and copious diarrhœa set in, for which he took a drink of hot whiskey. There were five thin, black stools, which he compared to liquid pitch, between nine and one o'clock. A violent ague attacked him at 10 A.M., when he was obliged to quit work and lie down in his quarters. During this chill he had great nausea and vomited violently four times, the first part of each ejection being thin and watery and the latter intensely green and bitter and thrown up with straining. There was also much abdominal pain between eleven and three o'clock. Admitted hospital 2 P.M. Temp. 102 1-5. ℞. Ipecac. gr. j at 2 and 4 P.M. No nausea during the afternoon but he had five stools, small, thin, reddish throughout as from blood, with minute yellow specks through them. There was no pain with these but they were very offensive in odor. 6 P.M.—Feels weak and prostrated but free from pain. Temp. 99 4-5. ℞. Ipecac. gr. x., opii gr. ss. at once and toward morning.

18th.—Slept well all night. The second dose was taken at 4 A.M. and there was no nausea with either. Feels much better but weak. Temp. 97 3-5. Arose after eating breakfast. Bowels not moved. Took ipecac. gr. v., opii gr. $\frac{1}{4}$, in pil. j at 2 P.M. Slightly chilly with feverish reaction and moderate perspiration. Temp. 6 P.M., 103 4-5. Repeat the pill at 9 P.M. and 4 A.M.

19th.—7 A.M., 97 4-5. Feels well and slept well all night. To have breakfast at 10 A.M. and another pill at 12 M. Temp. 1 P.M., 97 4-5. Dinner at 3 P.M. Temp. 6 P. M., 98. Another pill. The bowels not having moved since the first large dose of ipecacuanha, to take Rochelle salts at 8:30 P.M.

20th.—Bowels moved painlessly four times in the night, the first three motions being thin and yellow and the last natural in color and consistence. Temp. 7 A.M., 98 2-5; 1 P.M., 98; 6 P.M., 98. Took a five-grain pill at ten and two o'clock.

21st.—Temp. 7 A.M., 98 1-10; 1 P.M., 98 1-5; 6 P.M., 98 4-5. To take ipecac. gr. j every two hours, beginning at 9 A.M.

22d.—Temp. A.M., 98. Has had no feeling of ague since 18th, as above noted. Returned to quarters and directed to take a two-grain pill every two hours.

23d.—Feels well. Returned to duty with instructions to take one or two grains of ipecacuanha four or five times a day for three weeks, and to report the first indication of a chill.

26th.—Reported at sick-call claiming that he had a chill yesterday. It appears that on the evening of 23d he missed parade, and that he has been in the guard-house since that night. The troops were paid off a day or two before, and he has been drinking somewhat. It is probable that he did not take his pills regularly, if at all, and that the chill recurred as he states. Temp. 10 A.M., 98 2-5. Remanded to the guard and directed to take two grains every two hours.

27th.—Temp. A.M., 99 2-5. Says he took five doses yesterday, but that he had a chill and a high fever at night. Admitted hospital. ℞. Ipecac. gr. ij every two hours. A light chill in the afternoon. Temp. P.M., 101 2-5.

28th.—Temp. A.M., 98 3-5; 2 P.M., 99; P.M., 103 4-5. A still lighter chill this evening. Treatment continued and, 10 P.M., took ipecac. gr. v., opii gr. ¼.

29th.—Temp. A.M., 101 2-5; P.M., 99 4-5. Two grains every two hours. No chill.

30th.—Temp. A.M., 97 1-5; P.M., 98 3-5. 31st.—Temp. A.M., 97 3-5; P.M., 99 1-5. Treatment continued and no chill.

1st January, 1875.—Temp. A.M., 96 3-5; P.M., 101 2-5. Notwithstanding this fluctuation he has no disagreeable sensations.

2d.—Temp. A.M., 99 4-5; P.M., 101. Treatment continued. No chill.

3d.—Temp. A.M., 99. Returned to duty, and to take one grain every three hours for three weeks.

19th January.—Again reports sick. Claims to have taken the one-grain pills regularly, to include yesterday. Says he felt chilly on the afternoon of 16th; on 17th had no chill but a high fever; on 18th had a sharp chill 1:30 P.M., with a fever lasting all the afternoon. Temperature to-day: A.M., 97 4-5; 11:30, 97 1-5; 1 P.M., 97; 2:30, 96 4-5; 6, 98 4-5. Took ipecac. gr. xx., tr. opii *m.* xx. at 11:30 A.M., and soon fell asleep. There was no vomiting nor uneasiness, and at 8:30 P.M. took ipecac. gr. v., opii gr. ¼.

20th.—Took the same pill at 4 and 9 A.M. and 1 P.M. Temp. 7 A.M., 98 1-5; 1 P.M., 98 4-5.

21st.—Temp. A.M., 97 3-5; P.M., 98 1-5. Repeated pill at nine, one and half-past six o'clock. Slept well all night and felt well all day.

22d.—Temp. A.M., 97 4-5; P.M., 98 2-5. Same dose 1 and 6 P.M.

23d.—Temp. A.M., 97 4-5. To take two grains every two hours continuously. Duty.

26th January.—Is on extra-duty as striker in the blacksmith shop: that is, is employed at hard labor under marked changes of temperature. Reports, 9 A.M., having had a chill with high fever last night. Temp. 102 3-5; P.M., 98 2-5. Two-grain doses resumed. doses resumed.

27th.—Temp. A.M., 97 1-5; P.M., 101 2-5. ℞. Ipecac. gr. v., opii gr. ¼ at 9 A.M. and 1 P.M, and ipecac. gr. x. (without opium)

at 6 P.M. Had no chill, but toward evening felt warm and went to bed. No emesis.

28th.—Temp. A.M., 99 3-5; P.M., 98 3-5. Ipecac. gr. iij every three hours.

29th.—Temp. A.M, 97 3-5; P.M., 101. Treatment continued.

30th.—Temp. A.M., 98 3-5; P.M., 98 3-5. ℞. Ipecac. gr. x., op. gr. ss. 6 A.M. and three grains every three hours through the day.

31st.—Temp. A.M., 98 2-5; P.M., 98 3-5. ℞. Ipecac. gr. iij every three hours.

1st February.—Temp. A.M., 98. Duty. Relieved from extra-duty and returned to his company. To take small quantities of ipecacuanha regularly and at short intervals for a long time.

There was no further relapse in this case.

*(Continued in next number of Journal.)*

